# X-Band Single Chip Integrated Pulsed Electron Spin Resonance Microsystem

**DOI:** 10.1021/acs.analchem.4c02769

**Published:** 2024-08-27

**Authors:** Reza Farsi, Nergiz Sahin Solmaz, Mattéo Maury, Giovanni Boero

**Affiliations:** Institute of Electrical and Micro Engineering (IEM) & Center for Quantum Science and Engineering (QSE) École Polytechnique Fédérale de Lausanne (EPFL), Lausanne CH-1015, Switzerland

## Abstract

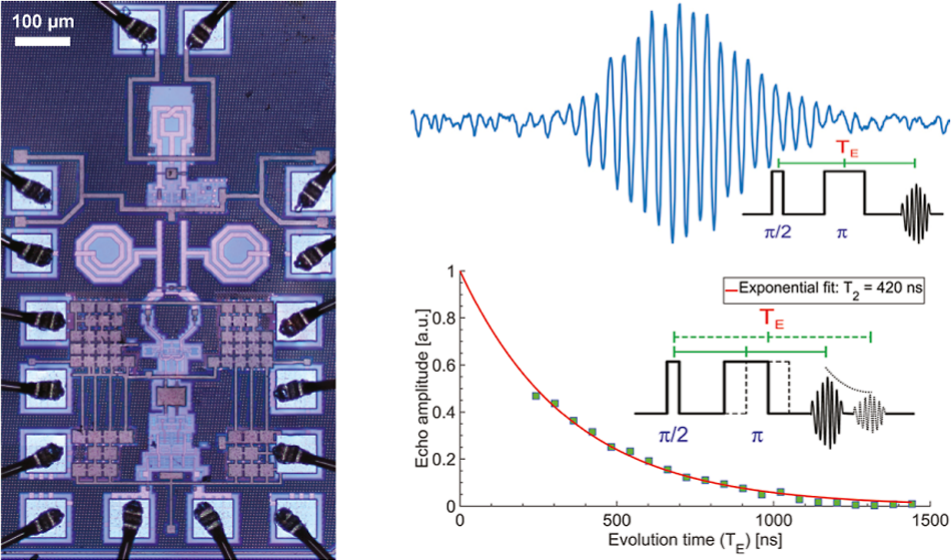

We report on the design and characterization of a single chip integrated pulsed electron spin resonance detector operating at 9.1 GHz. The microsystem consists of an excitation microcoil, a detection microcoil, a low noise microwave preamplifier, a mixer, and an intermediate frequency (IF) amplifier. The chip area is about 0.7 mm^2^. To exemplify its possible applications, we report the results of single pulse, Rabi nutation, Hahn echo, two echoes, Carr-Purcell, and inversion recovery echo experiments performed on 0.02 and 0.05 nL samples of α, γ-bisdiphenylene-β-phenylallyl (BDPA) and 1% BDPA in polystyrene (BDPA:PS) at room temperature. The measured spin sensitivity is about 8 × 10^7^ spins/Hz^1/2^ on a sensitive volume of about 0.1 nL. The microsystem power consumption is less than 100 mW, the radio frequency (RF) input bandwidth is 8.8 to 9.8 GHz, the IF output bandwidth is DC to 350 MHz, and the deadtime is less than 30 ns.

## Introduction

Electron spin resonance (ESR) spectroscopy finds a wide range of applications in various scientific disciplines, including chemistry, physics, biology, and materials science. It is especially valuable in the study of free radicals, transition metal ions, and defects in crystals, providing crucial information about their electronic and geometric structures.^1−4^ Since the first successful ESR signal observation by Zavoisky in 1944,^5^ the instrumentation for ESR spectroscopy has covered an increasingly broader range of operating frequencies and magnetic fields, sample volumes, temperatures, pressures, and excitation/detection methods.^1−4^

A promising approach for high sensitivity and low cost ESR spectroscopy on nanoliter and subnanoliter samples is the integration of the sensitivity relevant part of the spectrometer into a single chip having an area of less than 1 mm^2^.^6^ Several single chip integrated ESR microsystems have been reported in the last 15 years, with operating frequencies from 8 to 260 GHz and operating temperatures from 1.4 to 300 K. Previously reported single chip integrated ESR microsystems are based on continuous wave (CW),^6−16^ rapid scan,^17^ and pulsed excitations.^18,19^ The reported sensitivity of these sensors are in range from 10^7^ to 10^12^ spins/Hz^1/2^ depending on the operating frequency, temperature, and sensitive volume.

Methods based on pulsed excitation have been first implemented for the nuclear magnetic resonance (NMR) spectroscopy, where they proved to be far superior to CW excitation methods in terms of richness of information that can be extracted.^20^ Today, all commercial NMR systems for spectroscopy and imaging are based on pulsed excitations. The pulsed excitation is very commonly used also for ESR spectroscopy,^2^ although CW techniques are still used in many industrial and research applications. Pulsed techniques combined with miniaturized conducting and superconducting resonators have been used for ESR spectroscopy of nanoliter and subnanoliter samples.^21−32^

In ref (19) a single chip integrated pulsed ESR microsystem operating at K-band (24 GHz) is described, but no results of ESR experiments are reported. Another K-band (30 GHz) single chip integrated pulsed ESR microsystem is described in ref (18) where a pulsed approach based on the pulsed modulation of the excitation frequency (instead of the conventional pulsed modulation of the excitation amplitude) is demonstrated.

In this work, we report on a single chip integrated pulsed ESR microsystem operating at X-band (9.1 GHz). The pulsed approach adopted here is based on the pulse modulation of the amplitude of the microwave field, as in conventional pulsed ESR. The microsystem consists of a complete receiver chain including a detection microcoil, a low noise microwave preamplifier, a double-balanced mixer fed by an external local oscillator (LO), and an intermediate frequency (IF) amplifier. An additional microcoil is cointegrated on the same chip for excitation. The measured deadtime is less than 30 ns. A π/2 flip angle is obtained with a pulse length of 10 ns, corresponding to a Rabi frequency of 25 MHz. The measured spin sensitivity of the microsystem is about 8 × 10^7^ spins/Hz^1/2^ at room temperature on a sensitive volume of about 0.1 nL. The aim of the proposed microsystem is to allow for high sensitivity low cost pulsed ESR experiments on nanoliter and subnanoliter samples. Thanks to small area occupied on the chip by the complete receiver (about 0.7 mm^2^), this approach is suitable also for the realization of arrays of detectors for parallel (simultaneous) ESR spectroscopy of multiple samples.

## Experimental Section

### Chemicals

For the experiments, we used subnanoliter single crystals of α, γ-bisdiphenylene-β-phenylallyl (BDPA/benzene, 152,560, Sigma-Aldrich) and subnanoliter pieces of 1% BDPA in polystyrene (BDPA:PS). The 1% BDPA:PS sample is prepared using 1 g of polystyrene (PS, 331,651, Sigma-Aldrich) solid polymer doped with 10 mg of BDPA (i.e., 1% in weight) using 40 mL of chloroform (CHCl_3_) as solvent. The BDPA sample has a spin density of about 1.5 × 10^27^ spins/m^3^ and relaxation times *T*_1_ ≅ *T*_2_ ≅ 100 ns.^33^ The 1% BDPA:PS sample has a spin density of about 1.2 × 10^25^ spins/m^3^ and relaxation times *T*_1_ ≅ 25 μs and *T*_2_ ≅ 1 μs as determined by the experiments shown below. The spin density in 1% BDPA:PS is computed considering that PS has a density of about 1 g/cm^3^.

### Instrumentation

**Figure 1:**
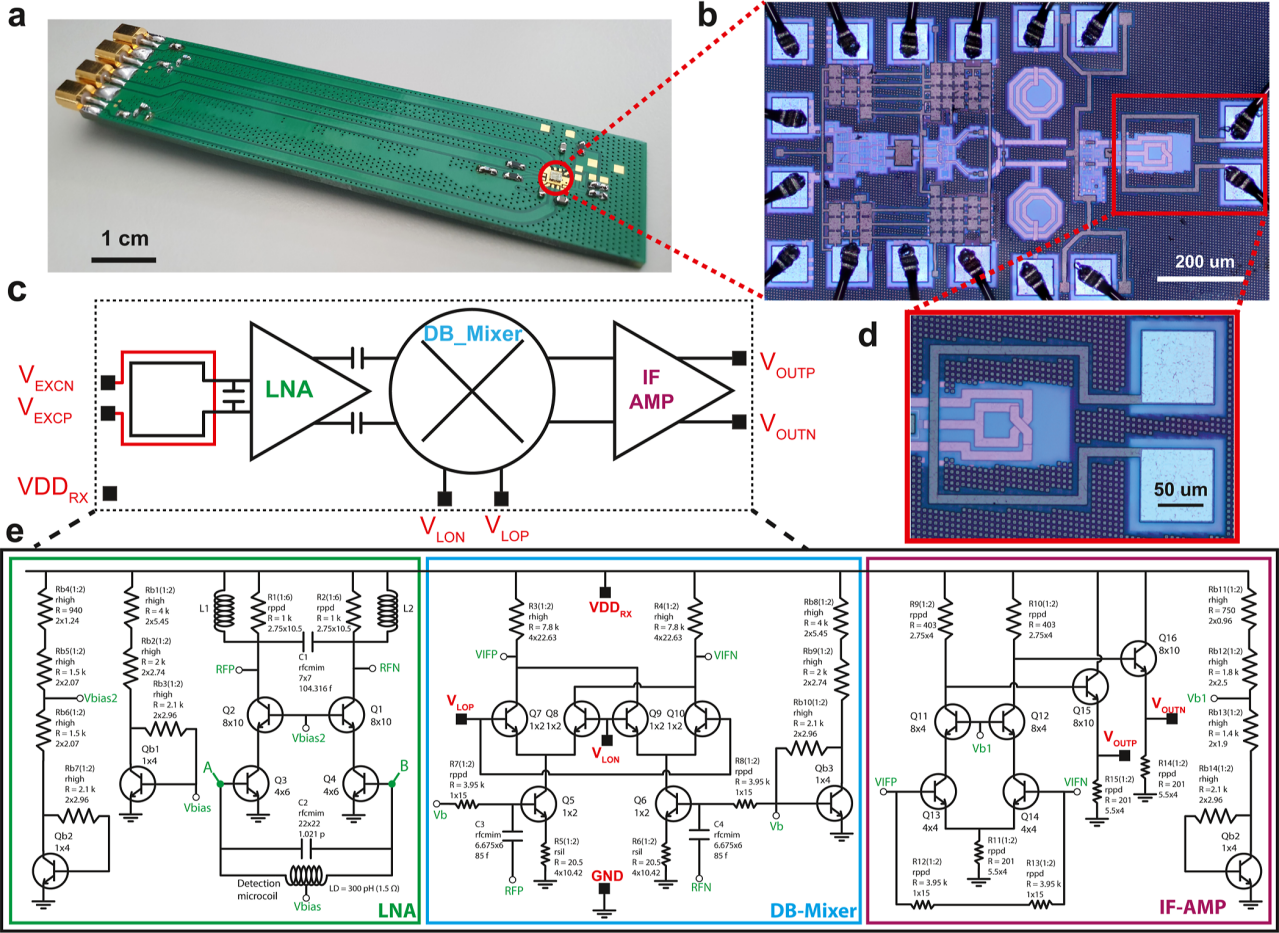
Single chip integrated pulsed ESR microsystem. (a) Printed circuit board (PCB) with the single chip integrated pulsed ESR microsystem. The chip is glued on the PCB and electrically connected by Au wire bonding. (b) Photograph of the pulsed ESR microsystem. The red rectangle indicates the excitation and detection microcoils. (c) Block diagram of the single chip pulsed ESR microsystem. VDD_RX_ is connected to several nets and the connections are not shown in the schematic for simplicity. VDD_RX_ is the supply voltage of all the blocks in the microsystem. (d) Photograph of the excitation and detection microcoils. (e) Transistor level schematic of the single chip pulsed ESR microsystem. The connections in red color are the input and output pads of the microsystem. The green connections are internal connections and connections to the biasing circuits. All transistors are the npn13G2 model of the IHP SG13G2Cu technology, having an emitter width of 70 nm and an emitter length of 900 nm.

The single chip integrated pulsed ESR microsystem consists of a complete receiver operating at X-band and a cointegrated excitation microcoil. The chip is manufactured in a 130 nm SiGe BiCMOS technology (IHP SG13G2Cu). The chip, shown in Figure 1b, has an area of 0.7 mm^2^ including the bonding pads and operates with a supply voltage VDD_RX_ in the range from 1 to 2.5 V and a corresponding current from 20 to 40 mA. Hence, the chip power consumption is in the range from 20 to 100 mW.

The electrical connections from the chip to the PCB are made by wedge–wedge Au bonding wires having a diameter of 20 μm. The excitation microcoil is a one turn square coil with an outer side of 180 μm and a width of 10 μm implemented with a 2.8 μm thick Al layer. The excitation microcoil is centered around the detection microcoil as shown in Figure 1d. Its inductance is 500 pH and its series resistance is 1.8 Ω. The excitation microcoil is bonded to a standard FR4 (1.6 mm thickness) PCB, using two 1.8 mm long 20 μm diameter Au wires. One side of the microcoil is grounded on the PCB and the other one is connected to a 50 Ω transmission line. No tuning/matching circuitry is used in the excitation path. The excitation frequency is set at the frequency with minimum power reflection, which is about 9.1 GHz.

The single chip pulsed ESR receiver consists of a detection microcoil, an X-band low noise amplifier (LNA), a double-balanced mixer (DB-mixer), and an IF amplifier. The receiver chain block diagram is shown in Figure 1c, and its detailed schematics are shown in Figure 1e. The detection microcoil is a two turns planar square inductor implemented using a 3.2 μm thick Cu layer. It has an outer side of 80 μm, a wire width of 10 μm, and spacing between wires of 5 μm. The detection microcoil has an inductance of 300 pH and a resistance of 1.5 Ω. The LNA has a 3 dB bandwidth of 2 GHz about 9.5 GHz (i.e., 8.5 to 10.5 GHz) and a maximum gain of 23 dB. The detection coil is resonated with a parallel 1 pF capacitance and amplifies the induced signal and noise by a factor of 10. The resulting gain at the output of the LNA including the microcoil resonance gain is 43 dB. This resonance determines an effective 3 dB bandwidth from 9.2 to 10 GHz for the ESR signal induced in the detection microcoil. The simulated input referred voltage noise at the coil ends is about 0.4 nV/Hz^1/2^, which includes the thermal noise of the detection microcoil and the equivalent input noise of the receiver electronics.

The excitation and detection microcoils are concentric and located in the same plane. Hence the microwave magnetic field produced by the excitation coil results in an induced microwave electromotive force and hence a microwave current in the detection microcoil. The detection microcoil is connected to the LNA, which has an input impedance (i.e., between pins A and B shown in Figure 1e) of about (400 Ω ∥ 200 fF). By considering the LNA input impedance and the detection resonator impedance, the microwave current induced in the detection microcoil produces a microwave magnetic field *B*_1_ which is approximately five times larger than the one produced by the current running into the excitation microcoil. The simulated microwave magnetic field *B*_1_ at the center of the detection microcoil is about 20 G with an amplifier output power of 10 W (see Supporting Information part B for details on these *B*_1_ simulations). The amplified signal by the LNA is frequency down converted by a double balanced mixer (DB-mixer) with an input bandwidth from 7 to 12 GHz. The DB-mixer has a maximum gain of 8 dB and an output bandwidth from DC to 500 MHz. In this design, the mixer LO signal are fed externally in the form of two sinusoidal signals with π phase difference. Both *V*_LOP_ and *V*_LON_ inputs are DC biased at a voltage *V*_DC,LO_ that can vary from 1 to 1.8 V. The next stage is a differential IF amplifier having a bandwidth from DC to 350 MHz and maximum gain of 14 dB. The output stage of the IF amplifier is capable to drive external electronics with 50 Ω input impedance. The receiver has maximum simulated total single output gain of 64 dB (VDD_RX_ = 1.8 V) and 76 dB (VDD_RX_ = 2.5 V), i.e., the differential gain is 70 and 82 dB, respectively.

The expected spin sensitivity can be computed as follows. The amplitude of the electromotive force induced in the detection coil by the spin precession after a π/2 flip angle, is^34^
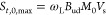
1where ω_L_ = γ*B*_0_ is the Larmor frequency (in rad/s), *B*_0_ is the static magnetic field (in *T*), γ is the gyromagnetic ratio (in rad/sT), *B*_ud_ is the component of the unitary magnetic field of the detection coil perpendicular to *B*_0_ (in T/A), *M*_0_ is the static magnetization (in A/m), and *V*_s_ is the sample volume (in m^3^). The static magnetization in the Curie law approximation *k_B_T* ≫ γ*ℏB*_0_ is given by
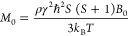
2where ρ is the number of spins per unit volume (in spins/m^3^), ℏ is the reduced Planck constant, *S* is the spin quantum number, *T* is the sample temperature (in *K*), and *k*_B_ is the Boltzmann constant. The spin sensitivity in time domain (in spins/Hz^1/2^) can be defined as *N*_min,*t*_ = *N*_s_/SNR_*t*_, where *N*_s_ is the number of spins in the sample, SNR_*t*_ is the signal-to-noise ratio (in 1/Hz^1/2^) defined as SNR_*t*_ = *S*_*t*,0,max_/*V*_n_, and *V*_n_ is voltage noise spectral density at the coil ends (in V/Hz^1/2^). Hence the spin sensitivity can be written as
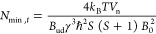
3

In the following, we compute the expected spin sensitivity for the ESR receiver described above. The unitary magnetic field in the center of the detection coil is *B*_ud_ ≅ 0.044 T/A (see Supporting Information, part B) and the voltage noise spectral density at the coil ends is *V*_n_ ≅ 0.4 nV/Hz^1/2^. From these values and assuming *S* = 1/2, *B*_0_ ≅ 0.32 T, γ ≅ 1.76 × 10^11^ rad/sT, *T* = 293 K, the expected spin sensitivity is *N*_min,*t*_ ≅ 3 × 10^7^ spins/Hz^1/2^.

### Setup for Pulsed ESR Measurements

**Figure 2:**
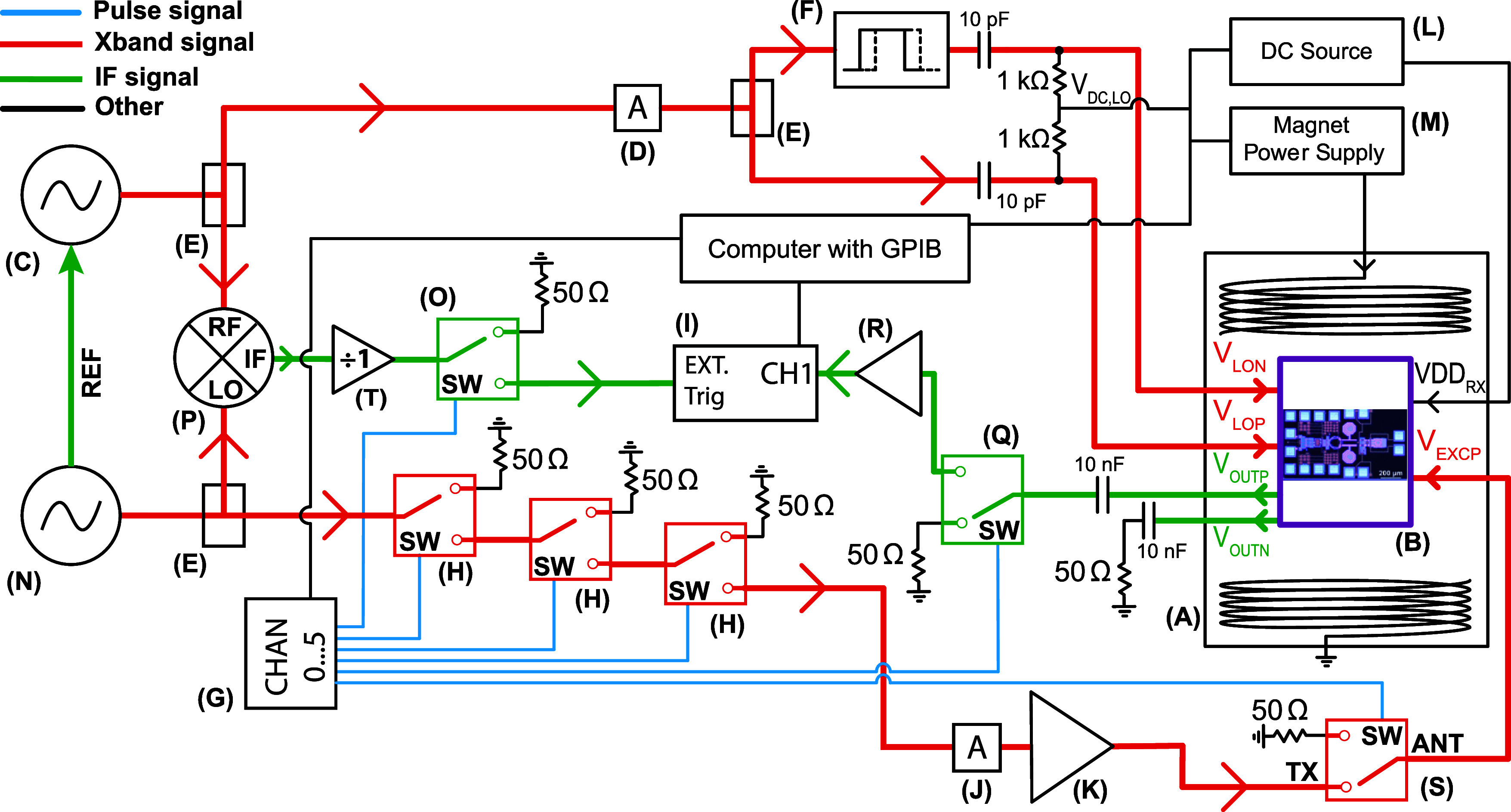
Set-up for the characterization of the single chip pulsed ESR microsystem. The red lines are the MW connections, the black lines are the GPIB connections and DC power lines, the blue lines are the LVTTL pulses, and the green lines are the IF signals. (A) Room temperature bore resistive electromagnet (0 to 2.1 T, Bruker). (B) Vacuum chamber connected to a turbomolecular pump (for the experiments in this work we worked in air). (C) Frequency synthesizer (Valon 5019). (D) 10 to 15 dB attenuator. (E) Power splitter (Mini-Circuits, ZX10-2-126). (F) Phase adjuster (Spectrum, LS-0012-2121). (G) High speed programmable pulse generator (SpinCore, PBESR-PRO-500-USB-RM-FP). (H) LVTTL SPDT nonreflective switch (Analog Devices, ADRF5024). (I) USB Oscilloscope (Keysight, P9242A). (J) 3 dB attenuator. (K) Power amplifier (MITEQ, AMF-6B-09501050-40P). (L) Power supplies. (M) Magnet power supply (0 to 150 A, Bruker). (N) Signal generator (Rohde & Schwarz SMR 20). (O) Switch (Mini-Circuits ZYSW-2-50DR). (P) Frequency mixer (Mini-Circuits ZX05-153S+). (Q) SPDT nonreflective switch (Mini-Circuits ZYSW-2-50DR). (R) LNA (Mini-circuits, ZFL1000-LN+). (S) SPDT nonreflective switch (Analog devices ADRF5142). (T) Frequency divider with TTL output (Valon 3010).

All experiments are performed at room temperature, in air, and in an ordinary laboratory without RF/MW shielding. The chip is glued on top of an FR4 PCB as shown in Figure 1a using conductive epoxy (Epo-Tek, H20E-FC). The PCB is placed in a 0 to 2 T resistive electromagnet. The connections from the PCB to the external electronics are realized with four coaxial cables for the RF/MW connections (*V*_LON_, *V*_LOP_, *V*_EXCP_, *V*_OUTP_) and three single pole wires for the DC connections (VDD_RX_, GND, *V*_DC,LO_). As shown in Figure 2, two microwave signal generators are used for the excitation and LO signals. The LO signal is split into two signals (*V*_LOP_ and *V*_LON_), one of them is π phase shifted before reaching the chip. *V*_LOP_ and *V*_LON_ signals are separately biased using two 1 kΩ resistors soldered on PCB as shown in Figure 2. On the excitation path, three switches (*H*) are implemented to pulse modulate the sinusoidal CW output of the signal generator (*N*). These switches are controlled by a multichannel programmable pulse generator (*G*) which produces LVTTL (0–3.3 V) pulses having a minimum length of 6 ns. The use of three switches in series allows to obtain an isolation of about 100 dB. A power switch (*S*) is used after the amplifier to further increase the isolation and to reduce the amplifier noise delivered to the excitation coil during the detection time of the ESR signal. In the IF signal path, the switch (*Q*) prevents the saturation of the external IF amplifier (*R*), avoiding a significant increase of the deadtime. The two microwave generators are frequency locked using the same 10 MHz reference signal. This assures a fixed frequency difference between the two generators (in our experiments 200 MHz). To allow for phase coherent time domain averaging of the ESR signals, the acquisition is triggered by the IF signal obtained by mixing the two generators using the mixer (*P*). This IF signal is shaped using the TTL output of a divider (*T*) and it passes through a switch (*O*) before reaching the trigger input of the data acquisition board (*I*). This allows to set the start of the data acquisition at the desired time with respect to the pulse sequence. In order to characterize the MW and IF bandwidth of the receiver, we applied a CW signal to the excitation microcoil. The 3 dB microwave bandwidth of the receiver is from 8.8 to 9.8 GHz. The 3 dB IF bandwidth of the receiver is DC to 350 MHz. The measured power consumption is less than 100 mW for a VDD_RX_ of 2.5 V. The maximum AC swing at the outputs pins *V*_OUTP_ and *V*_OUTN_ is 400 mV peak-to-peak about a DC level of 1.15 V. All these experimental results are in agreement with the simulated values reported above. However, the measured output noise of the receiver is 250 nV/Hz^1/2^ from 1 to 300 MHz whereas the simulated value is 2.8 μV/Hz^1/2^. The lower measured output noise of the microsystem is caused by an overall gain lower than the simulated value of 76 dB (for a single output at 2.5 V, see above). The overall gain cannot be measured directly because the input stage of the receiver chain cannot be accessed. However, the overall gain can be estimated with pulsed ESR measurements on samples of known spin density and volume. In particular, we performed measurements with a single crystal of BDPA having a volume of about 1.9 × 10^–14^ m^3^ and a spin density of about 1.5 × 10^27^ spins/m^3^. The expected signal amplitude at the input, computed from eq 1, is 0.3 mV. The measured output signal is about 0.1 V (see Experiments with BDPA), hence the overall gain is about 50 dB, i.e., about 20 dB lower than expected. The lower measured gain is consistent with the lower measured noise mentioned above. For the moment, we have not found a convincing explanation for this significant overall gain reduction. Further details on the simulation results of the receiver electronics are reported in the Supporting Information part C.

## Results and Discussion

In order to exemplify the versatility of the proposed ESR microsystem, we performed several conventional pulsed ESR experiments such as single pulse, Rabi nutation, Hahn echo, two echoes, Carr-Purcell (CP) echoes, and inversion recovery echo experiments. To reduce the off-resonance effects, all experiments are performed with an excitation frequency equal to the Larmor frequency, specifically of about 9.1 GHz in the applied static magnetic field of about 325 mT. As mentioned above, the LO frequency *f*_LO_ is 200 MHz above the excitation frequency.

As samples, we use BDPA and 1% BDPA:PS, as these two samples represent a variety of scenarios in terms of relaxation times, spin densities, and inhomogeneously/homogeneously broadened lines.

### Experiments with BDPA

**Figure 3:**
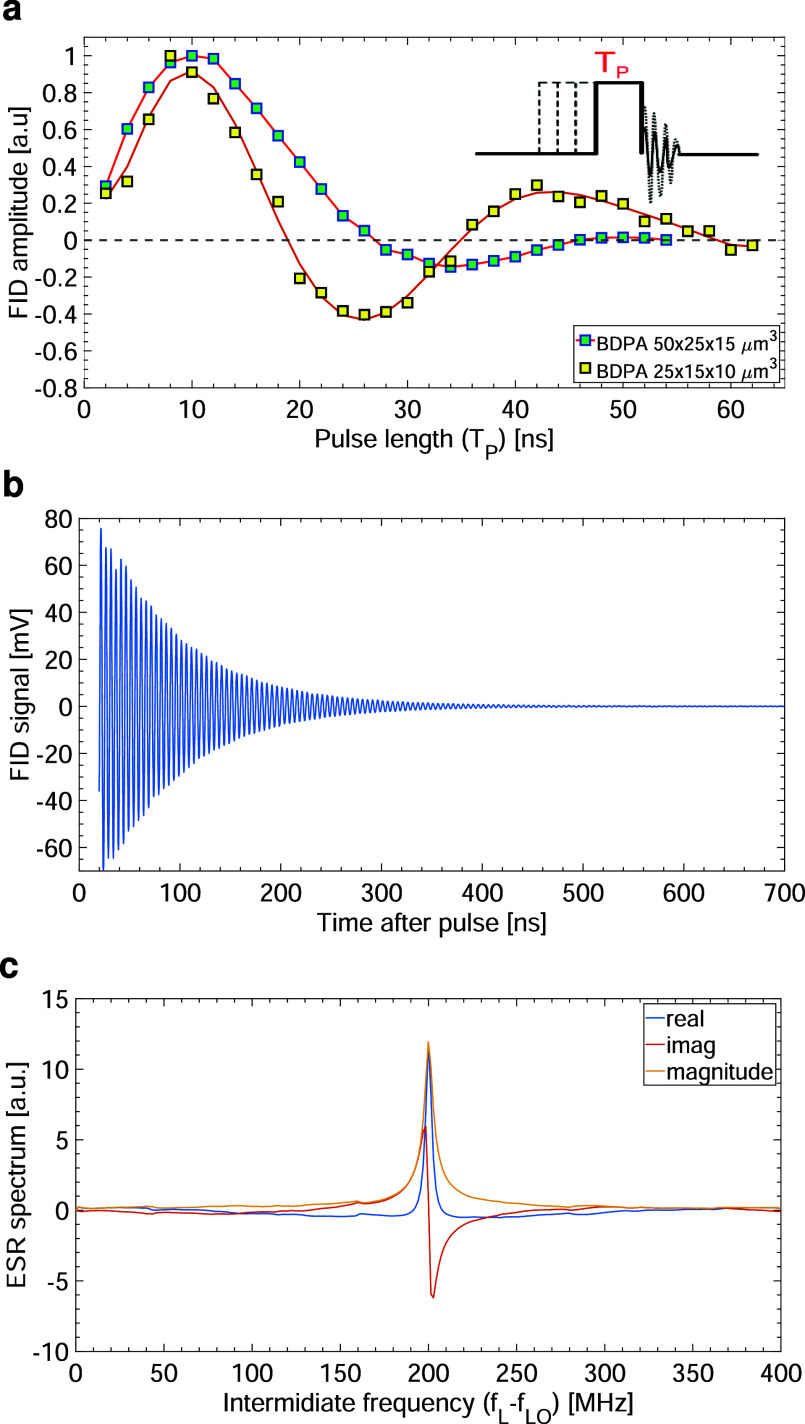
Pulsed ESR experiments with BDPA crystals. (a) Amplitude of the FID signal at the beginning of the decay as a function of the excitation pulse length *T*_P_ for two BDPA crystals having different volumes, 50 × 25 × 15 μm^3^ and 25 × 15 × 10 μm^3^, respectively. (b) Time domain FID signal of the larger BDPA crystal after 20 ns deadtime. (c) Fast Fourier transform of the time domain FID signal. The excitation pulse is 12 ns, the repetition time is 12 μs, and the number of averaging is 65,000 (i.e., the effective measurement time is about 0.8 s). The excitation frequency is 9.1 GHz and the static magnetic field is about 325 mT.

In Figure 3 are reported experiments performed with two crystals of BDPA having a volume of about 50 × 25 × 15 μm^3^ and 25 × 15 × 10 μm^3^. The measured deadtime after the excitation pulse is approximately 5 ns, but it can increase up to 25 ns depending on the applied excitation power and pulse length. In these experiments, the external IF amplifier [device (*R*) in Figure 2] is not used. In Figure 3a, the normalized amplitude of the free induction decay (FID) signal at the beginning of the decay as a function of the excitation pulse length *T*_P_ with an excitation power of 36 dBm at the output of the power amplifier is reported. From this measurement, we obtain a Rabi nutation frequency (Ω/2π) of about 25 MHz. Hence, the microwave magnetic field *B*_1_ is Ω/γ ≅ 9 G, where γ ≅ 1.76 × 10^11^ rad/sT is the electron gyromagnetic ratio, in good agreement with the simulated value of 20 G discussed above. As shown in Figure 3b, the decay time of the Rabi nutation curve of a smaller 25 × 15 × 10 μm^3^ sample is significantly longer. As previously mentioned, most of the microwave magnetic field *B*_1_ is produced by the induced current in the detection coil. As a result, samples having a volume similar or larger than the detection microcoil are exposed to a more non uniform *B*_1_, which explains the shorter decay time of the corresponding Rabi nutation curve. The decay time of the nutation curve for the smaller sample is mainly caused by the spin relaxation which is not entirely negligible during the excitation time. In Figure 3b,c, the FID signal of the larger crystal after 20 ns deadtime and its Fourier transform obtained with a single π/2 pulse of 12 ns and an excitation power of 38 dBm is reported. All experiments are performed with a repetition time *T*_r_ = 12 μs (i.e., 100 times longer than *T*_1_), and the number of averaging is 65,000 (i.e., the effective measurement time is about 0.8 s).

The measured voltage noise spectral density at the output of the chip is about 250 nV/Hz^1/2^ in the IF frequency range from 1 to 300 MHz. Considering the density of spins in the sample (1.5 × 10^27^ spins/m^3^), the sample volume (1.9 × 10^–14^ m^3^), the voltage noise spectral density (250 nV/Hz^1/2^), and the signal amplitude at the beginning of the decay (0.08 V), the experimental spin sensitivity of the single chip pulsed ESR microsystem is 8 × 10^7^ spins/Hz^1/2^, i.e., less than three times worse than the expected value of 3 × 10^7^ spins/Hz^1/2^ computed above (see Supporting Information part A for further details on the spin sensitivity).

### Experiments with 1% BDPA:PS

**Figure 4:**
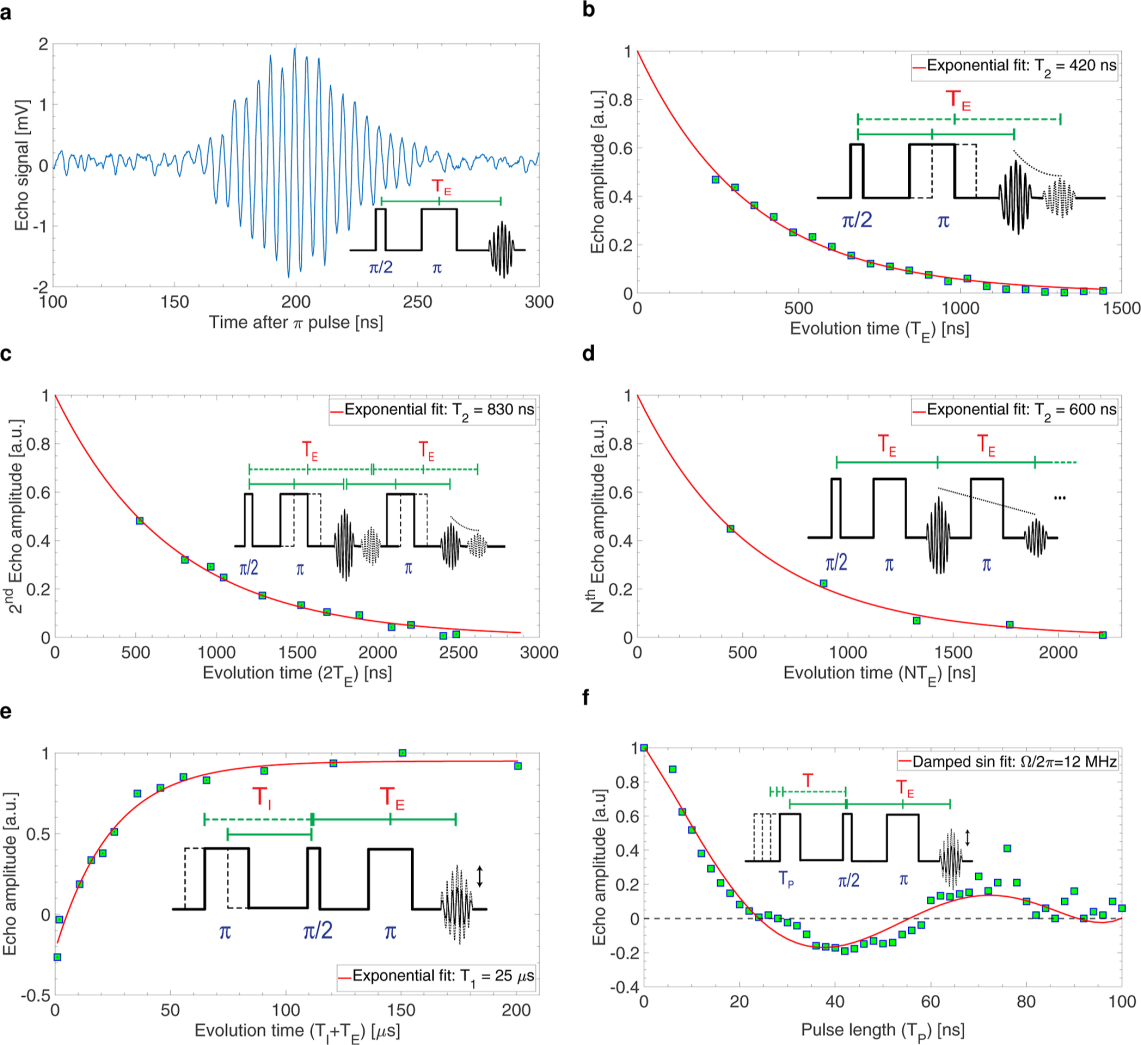
Pulsed ESR experiments with a 1% BDPA:PS sample. All the measurements are performed on a 50 × 50 × 15 μm^3^ 1% BDPA:PS sample at room temperature. The excitation frequency is 9.1 GHz and the static magnetic field is about 325 mT. The repetition time is *T*_r_ = 1200 μs (i.e., about 50 times longer than *T*_1_), and the number of averaging is 1.3 M (i.e., the effective measurement time is about 1600 s). The pulse sequence for each measurement is shown as inset. The dashed lines indicate the parameter that is varied during the experiment. *T*_P_ is the pulse length. *T*_E_ is the echo time, which is the time between the π/2 pulse and the center of the echo in the Hahn and CP echoes sequences. *T*_I_ is the inversion time, which is the time between the π pulse and the π/2 pulse in the inversion recovery Hahn echo sequence. *T* is the time between the *T*_P_ pulse and the π/2 pulse in the Rabi nutation Hahn echo sequence. (a) Measured spin echo with *T*_E_ = 400 ns. (b) Amplitude of the Hahn echo with *T*_E_ from 200 to 1500 ns. The transversal spin relaxation time measured with this Hahn echo sequence is *T*_2_ ≅ 420 ns. (c) Amplitude of the second echo of the two echoes sequence with *T*_E_ from 250 to 1500 ns. The transversal spin relaxation time measured with this two echoes sequence is *T*_2_ ≅ 830 ns. (d) Amplitude of the *N*th echo of the CP echoes sequence, where *N* is the number of π pulses applied after the π/2 pulse. *T*_E_ is set to 440 ns during the whole experiment. The distance between the π pulses are also constant and equal to *T*_E_ = 440 ns. The transversal spin relaxation time measured with this CP echoes sequence is *T*_2_ ≅ 600 ns. (e) Amplitude of the Hahn echo measured at a time *T*_I_ + *T*_E_ after the inversion π pulse. *T*_I_ is varied from 2 to 200 μs and *T*_E_ = 600 ns for all experiments. The longitudinal spin relaxation time measured with this inversion recovery sequence is *T*_1_ = 25 μs. (f) Amplitude of Hahn echo measured *T* = 6 μs + *T*_E_ = 600 ns after the pulse of length *T*_P_, which is varied from 0 to 100 ns. The Rabi frequency measured with this nutation Hahn echo sequence is Ω/2π ≅ 12 MHz.

In Figure 4 are reported the results of Hahn echo, two echoes, CP echoes, inversion recovery echo, and Rabi nutation echo experiments performed on a sample of 1% BPDA:PS having a volume of about 50 × 50 × 15 μm^3^ (i.e., slightly larger than the volume of the BDPA sample measured above). In these experiments, the external IF amplifier [device (*R*) in Figure 2] is used to amplify the signal above the output discretization limit of the digitizer (I). The use of the external IF amplifier increases the effective dead time for 25 to 100 ns. All experiments are performed with a repetition time *T*_r_ = 1200 μs (i.e., about 50 times longer than *T*_1_), and the number of averaging is 1.3 M (i.e., the effective measurement time is about 1600 s).

Figure 4a shows the time domain signal obtained with a Hahn echo experiment (π/2 – *T*_E_/2 – π) with echo time *T*_E_ = 400 ns. In Figure 4b is reported the amplitude of the echo obtained with Hahn echo experiments performed with echo times *T*_E_ from 200 to 1500 ns. In Figure 4c is reported the amplitude of the echo obtained with the two echoes sequence (π/2 – *T*_E_/2 – π – *T*_E_ – π) with echo times *T*_E_ from 250 to 1500 ns. In Figure 4d is reported the amplitude of the echo obtained with the CP echoes sequence (π/2 – *T*_E_/2 – π – *T*_E_ – π – *T*_E_ −···) performed with *T*_E_ = 440 ns. From the Hahn echo, two echoes, and CP echoes experiments *T*_2_ values of 0.42 μs, 0.83 and 0.6 μs are extracted, respectively. In Figure 4e are reported the results of inversion recovery echo experiments (π – *T*_I_ – π/2 – *T*_E_/2 – π), performed with inversion time *T*_I_ from 2 to 200 μs and *T*_E_ = 600 ns. From the exponential fit of the obtained curve, a relaxation time *T*_1_ ≅ 25 μs is obtained. In Figure 4f are reported the results of a Rabi nutation echo experiment (*T*_P_ – *T* – π/2 – *T*_E_/2 – π), performed with *T*_P_ from 0 to 100 ns, *T* = 6 μs, and *T*_E_ = 600 ns. From these measurements, we obtain a Rabi nutation frequency of about 12 MHz with an excitation power of 38 dBm. This value is smaller than the one obtained with the single pulse Rabi nutation experiment on a smaller BDPA sample reported above (about 25 MHz). This difference is presumably due to the larger *B*_1_ inhomogeneity and the use of a time *T* not much shorter than *T*_1_.

## Conclusions

In this work, we demonstrated, for the first time, the integration on a single chip of about 0.7 mm^2^ of a pulsed ESR microsystem consisting of an excitation microcoil, a detection microcoil, a low noise microwave amplifier, a double-balanced mixer, and an IF amplifier. The single chip ESR microsystem operates in the frequency band from 8.8 to 9.8 GHz and has a power consumption of less than 100 mW. The IF bandwidth is from DC to 350 MHz. We report single pulse, Rabi nutation, Hahn echo, two echoes, Carr-Purcell, and inversion recovery echo experiments on BDPA and 1% BDPA:PS at room temperature. The measured spin sensitivity is about 8 × 10^7^ spins/Hz^1/2^ on a sensitive volume of about 0.1 nL.

In the near future, we aim to extend the single chip pulsed ESR approach demonstrated here to low temperature and higher frequency as well as for array of detectors on the same chip for parallel (simultaneous) ESR spectroscopy of multiple samples. The integration of the microwave source, the switches, and the excitation pulse amplifier will be also investigated. Some of these extensions have been already reported for single chip CW ESR microsystems (oscillators and arrays) and pulsed NMR systems (pulse amplifiers and switches).^6−15^ The external (i.e., non integrated) electronics will be improved to allow for the control of the phase of the excitation pulses.

## Supporting Information Available

Supplementary: Further simulation results, more detailed discussions on the excitation magnetic field, and a brief discussion about measured spin sensitivity in both time and frequency domains (PDF)
