# Supplementary material for: X-Band Single Chip Integrated Pulsed Electron Spin Resonance Microsystem
Source: Anal Chem. 2024 Aug 27;96(36):14516–23. doi: 10.1021/acs.analchem.4c02769 (PMC11391408; doi:10.1021/acs.analchem.4c02769)
Supplement: Supplementary file 1 — ac4c02769_si_001.pdf [file ac4c02769_si_001.pdf]

Supporting Information
X-band single chip integrated pulsed electron spin resonance microsystem

Reza Farsi[1,*], Nergiz Sahin Solmaz[1,*], Mattéo Maury, Giovanni Boero

Institute of Electrical and Micro Engineering (IEM) & Center for Quantum Science and Engineering (QSE)

École Polytechnique Fédérale de Lausanne (EPFL), CH-1015 Lausanne, Switzerland

Contents

A Spin sensitivity in the time and frequency domains **S-2**

B Determination of the excitation microwave magnetic field B_1 **S-4**

C Simulation of the receiver gain and noise **S-5**

[*]Corresponding authors

Email addresses: reza.farsi@epfl.ch (Reza Farsi), nergiz.sahin@epfl.ch (Nergiz Sahin Solmaz)

[1]These authors contributed equally to the work.

A. Spin sensitivity in the time and frequency domains

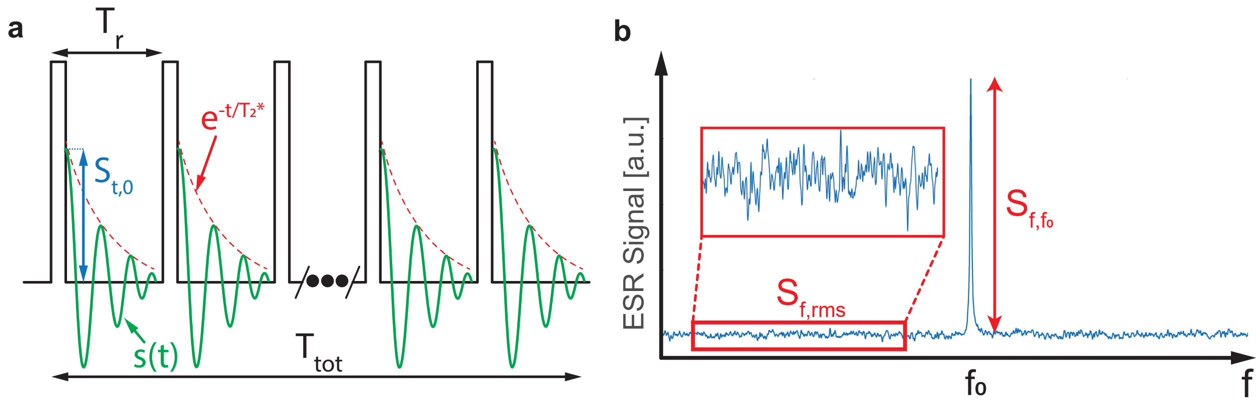

Figure S1: Illustration of the experiment to evaluate the spin sensitivity. **a** The experiment consists of a series of microwave pulses with a repetition time T_r, for a total duration of the experiment T_{tot}. After each pulse the resulting free induction decay signal is digitized for a duration $T \cong T_r$. **b** Average of the spectra obtained by Fourier transform (FT) of the the time domain free induction decay signals.

Let's consider a simple pulsed ESR experiment consisting of a series of microwave pulses with a repetition time T_r, for a total duration of the experiment T_{tot} (see Fig. S1). After each pulse the resulting free induction decay signal is frequency downconverted and acquired for a duration $T \cong T_r$. The acquired signal after each pulse can be written as

$$x(t) = s(t) + n(t) = S_{t,0}\cos(2\pi f_0 t)e^{-t/T_2^*} + n(t) \tag{1}$$

where $S_{t,0}$ is the signal amplitude at $t = 0$ (in V), $f_0 = f_L - f_{LO}$ is the frequency of the acquired signal (in Hz), f_{LO} is the local oscillator frequency used to downconvert the ESR signal (in Hz), $f_L = (1/2\pi)\omega_L$ is the Larmor frequency (in Hz), $\omega_L = \gamma B_0$ is the Larmor angular frequency (in rad/s), B_0 is the static magnetic field (in T), γ is the gyromagnetic ratio (in rad/sT), and T_2^* is the effective decay time of the free induction decay (in s). For homogeneously broadened lines $T_2^* = T_2$ whereas for inhomogeneously broadened lines $T_2^* \leq T_2$, where T_2 is the transversal relaxation time (in s). For a given values of T_r and T_1, the ESR signal amplitude $S_{t,0}$ is maximized with a flip angle $\beta_{opt} = \arccos(e^{-T_r/T_1})$ [1], where T_1 is the longitudinal relaxation time (in s). With this optimum angle, usually called Ernst angle, the ESR signal amplitude $S_{t,0}$ is

$$S_{t,0} = S_{t,0,max}\sqrt{\frac{1-e^{-T_r/T_1}}{1+e^{-T_r/T_1}}} \tag{2}$$

where $S_{t,0,max}$ is the amplitude of the ESR signal at $t = 0$ obtained with a $\pi/2$ pulse and $T_r \gg T_1$ [1]. As discussed in the main text, $S_{t,0,max} = \omega_L B_{ud} M_0 V_s$, where B_{ud} is the component of the unitary magnetic field of the detection coil perpendicular to B_0 (in T/A), M_0 is the static magnetisation (in A/m), and V_s is the sample volume (in m^3).

Prior to Fourier transform, the signal $x(t)$ is multiplied by a time domain filter function, usually consisting of an exponential function $e^{-t/\tau}$. The time domain filtered signal $x_F(t)$ can be written as

$$x_F(t) = x(t)e^{-t/\tau} = s_F(t) + n_F(t) = S_{t,0,max}\sqrt{\frac{1-e^{-T_r/T_1}}{1+e^{-T_r/T_1}}}\cos(2\pi f_0 t)e^{-t/T_2^*}e^{-t/\tau} + n(t)e^{-t/\tau} \tag{3}$$

The filtered time domain signal $x_F(t)$ is subsequently Fourier transformed. The maximum of the real part of the Fourier transformed signal $\mathfrak{Re}(s_F(f))$ occurs at $f = f_0$ and its amplitude is

$$S_{f,f_0} = S_{t,0,max}\sqrt{\frac{1-e^{-T_r/T_1}}{1+e^{-T_r/T_1}}}\left(1-e^{-T_r(\frac{1}{T_2^*}+\frac{1}{\tau})}\right)\left(\frac{1}{T_2^*}+\frac{1}{\tau}\right)^{-1} \tag{4}$$

The root mean square (rms) value of the noise fluctuations in $\mathfrak{Re}(n(f))$ is

$$S_{f,rms} = V_n\sqrt{\frac{\tau}{2}(1-e^{-2T_r/\tau})} \tag{5}$$

where V_n is the voltage spectral density of the time domain noise given by

$$V_n = \sqrt{PSD} = \sqrt{\frac{1}{T_r}|n(f)|^2} \qquad \left[\frac{\mathrm{V}}{\sqrt{\mathrm{Hz}}}\right] \tag{6}$$

where PSD is the power spectral density, $n(f)$ is the Fourier transform of the original time domain noise $n(t)$ (i.e., the time domain noise before time domain filtering). In the total experimental time T_{tot} the number of acquired spectra is T_{tot}/T_r. By averaging the obtained spectra we can calculate

$$S_{f,f_0,T_{tot}} = S_{f,f_0} = S_{t,0,max}\sqrt{\frac{1-e^{-T_r/T_1}}{1+e^{-T_r/T_1}}}\left(1-e^{-T_r\left(\frac{1}{T_2^*}+\frac{1}{\tau}\right)}\right)\left(\frac{1}{T_2^*}+\frac{1}{\tau}\right)^{-1} \tag{7}$$

$$S_{f,rms,T_{tot}} = S_{f,rms}\sqrt{\frac{T_{tot}}{T_r}} = V_n\sqrt{\frac{\tau}{2}(1-e^{-2T_r/\tau})}\sqrt{\frac{T_r}{T_{tot}}} \tag{8}$$

The signal-to-noise ratio in the frequency domain can be defined as

$$SNR_{f,T_{tot}} = \frac{S_{f,f_0,T_{tot}}}{S_{f,rms,T_{tot}}} = SNR_t\frac{\sqrt{\frac{1-e^{-T_r/T_1}}{1+e^{-T_r/T_1}}}\left(1-e^{-T_r\left(\frac{1}{T_2^*}+\frac{1}{\tau}\right)}\right)}{\sqrt{\frac{\tau}{2}(1-e^{-2T_r/\tau})}\left(\frac{1}{T_2^*}+\frac{1}{\tau}\right)}\sqrt{\frac{T_{tot}}{T_r}} \qquad [-] \tag{9}$$

where SNR_t is the time domain signal-to-noise ratio defined as

$$SNR_t = \frac{S_{t,0,max}}{V_n} \qquad [\sqrt{\mathrm{Hz}}] \tag{10}$$

The signal-to-noise ratio in the frequency domain per unit of time (i.e., in a measurement time $T_{tot} = 1$ s) is

$$SNR_f = SNR_{f,T_{tot}}\sqrt{\frac{1}{T_{tot}}} = SNR_t\frac{\sqrt{\frac{1-e^{-T_r/T_1}}{1+e^{-T_r/T_1}}}\left(1-e^{-T_r\left(\frac{1}{T_2^*}+\frac{1}{\tau}\right)}\right)}{\sqrt{\frac{\tau}{2}(1-e^{-2T_r/\tau})}\left(\frac{1}{T_2^*}+\frac{1}{\tau}\right)}\sqrt{\frac{1}{T_r}} \qquad [\sqrt{\mathrm{Hz}}] \tag{11}$$

It can be shown that the SNR_f is maximized for $T_1 \cong T_2^* \cong \tau$ and $T_r \cong 1.26T_2^*$. In these optimal conditions

$$SNR_{f,opt} \cong \frac{1}{2}SNR_t \tag{12}$$

Hence, with the definitions given above, the signal-to-noise ratio per unit of time in the frequency domain SNR_f in optimal conditions differs from the signal-to-noise ratio in the time domain SNR_t only by a factor of 2.

The spin sensitivity in the time and frequency domain can be defined as

$$N_{min,f} = \frac{N_s}{SNR_f} = \frac{\rho V_s}{SNR_f} \qquad \left[\frac{\mathrm{spins}}{\sqrt{\mathrm{Hz}}}\right] \tag{13}$$

$$N_{min,t} = \frac{N_s}{SNR_t} = \frac{\rho V_s}{SNR_t} \qquad \left[\frac{\mathrm{spins}}{\sqrt{\mathrm{Hz}}}\right] \tag{14}$$

where N_s is the number of spins in the sample, V_s is the sample volume (in m^3), and ρ is the density of spins in the sample (in spins/m^3).

The previous two equations allow to compare ESR systems when the time domain or frequency domain spin sensitivity are given. If the time domain spin sensitivities are given, no experimental parameter are required to compare different systems. On the other hand, if the frequency domain spin sensitivity is given, the experimental parameters T_1, T_2^*, τ, T_r are required for a fair comparison. In this sense, the time domain spin sensitivity is somehow a better way to characterize the spin sensitivity of a system because it does not depend on the specific properties of the sample. However, for samples with $T_2^* \ll T_1$, the time domain spin sensitivity is a too optimistic estimation of the effectively achievable spin sensitivity per unit of time for that sample. Note that the signal-to-noise ratio SNR_f is defined as the signal amplitude divided by the rms value of the noise. However, in order to clearly distinguish a signal from the noise, the signal amplitude should be about 2 to 3 times larger than the rms value of the noise. This means that the value of the spin sensitivity computed with this definition of SNR_f is about 2 to 3 times better than the one effectively achievable. In comparing the spin sensitivities, it is then important to consider also the way the SNR_f is defined. For example, in [1] a factor 2 is introduced.

Using the previous equations we can now compare the spin sensitivity obtained with the single chip pulsed ESR microsystem reported in this work with those obtained in previous pulsed ESR works with miniaturized

detectors having sensitive volumes in the nL range. We will limit the comparison to experiments performed at room temperature. Let's start by converting the time domain spin sensitivity obtained in our work into the one in the frequency domain. The measured time domain spin sensitivity is $N_{min,t} \cong 8 \times 10^7$ spins/Hz$^{1/2}$. This spin sensitivity is obtained with a 0.02 nL BDPA sample having $T_1 \cong T_2 \cong T_2^* \cong 100$ ns. Since $(T_1 \cong T_2^* \cong \tau \cong T_r)$, $N_{min,f} \cong N_{min,f,opt} \cong 1.6 \times 10^8$ spins/Hz$^{1/2}$.

The best room temperature spin sensitivities reported to date are probably those in Ref.[2], where a frequency domain spin sensitivity $N_{min,f} \cong 5.2\times10^6$ spins/Hz$^{1/2}$ on a 0.25 nL sample and of $N_{min,f} \cong 4.5\times10^7$ spins/Hz$^{1/2}$ on a 1.2 nL sample are demonstrated. These experiments are performed with a sample of DFT-$(SO_3)_3$ having $T_1 \cong 8.5$ μs and $T_2 \cong 1.5$ μs. The T_2^* value is not reported, but from the observation of the linewidth it is probably in the order of 1 μs. The spin sensitivity is evaluated with Hahn echo experiments. Due to the difference between T_1 and T_2^*, the frequency domain spin sensitivity normalized to samples having $T_1 \cong T_2^*$ would improve by a factor of about $(T_1/T_2^*)^{1/2} \cong 3$. These experiments are performed at about 35 GHz at room temperature. From the comparison with the two examples above (and assuming that we interpret the reported spin sensitivities properly), we conclude that the single chip pulsed ESR microsystem of this work has a spin sensitivity which is up to a factor 80 times worse, partially due to the lower operating frequency f_L and static magnetic field B_0.

B. Determination of the excitation microwave magnetic field B_1

The transmitter circuitry is sketched in Fig. S2a. The power amplifier output stage is modeled by the voltage source V_S with 50 Ω output impedance. Since the power amplifier delivers a power of 10 W, $V_S \cong 63$ V. The power amplifier is connected to the PCB by a coaxial cable having a length of about 60 cm and losses of about 3 dB. The single chip pulsed ESR microsystem is glued on a printed circuit board (PCB) and electrical connected by wire bonding. The connections on the PCB are modeled by a parasitic capacitor C_{PCB} representing the parasitic capacitance of the bonding pad on the PCB, an inductor L_{wb} and a resistor R_{wb} representing the Au bonding wire having a length of 1.8 mm and diameter of 20 μm, a parasitic capacitor C_{chip} to take into account the 80 × 80 μm^2 bonding pad on the chip, an inductor L_e and a resistor R_e modeling the square excitation microcoil with 180 μm outer side. The other end of the excitation microcoil is connected to the bonding pad on the chip and with a bonding wire to ground on the PCB. In Fig. S2a, it is also sketched the first part of the integrated receiver chain. The detection microcoil is modeled with a two turns square coil having an outer side of 80 μm, an inductance L_d, and a resistance R_d. The detection microcoil is connected in parallel to a tuning capacitor C_t. The input impedance of the LNA is modeled as a resistor R_{LNA} in parallel with a capacitor C_{LNA}, as obtained by Cadence Virtuoso simulations.

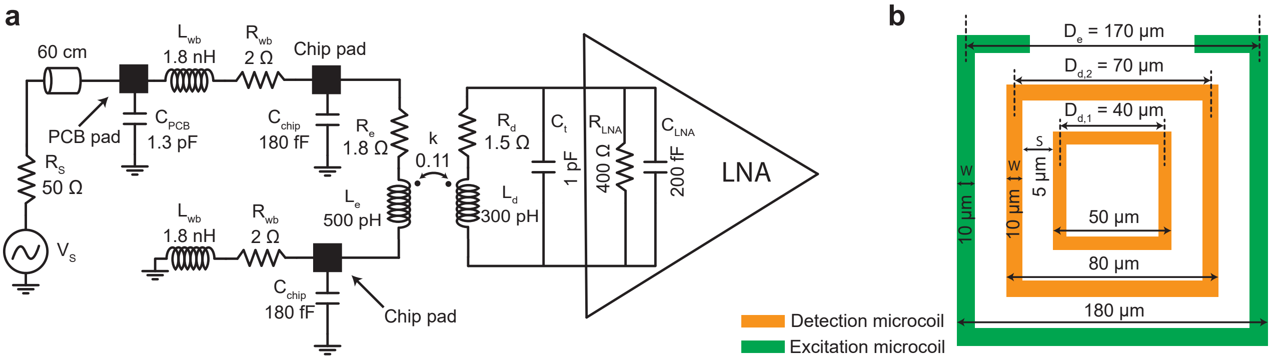

Figure S2: Electrical model for the determination of the excitation microwave magnetic field B_1 **a** Approximate electrical model of the transmitter circuitry and of the front-end of the receiver circuitry. **b** Approximate geometrical model of the excitation and detection microcoils (not to scale).

The coupling factor between the excitation and the detection microcoils is approximately given by

$$k \cong \frac{L_{ed}}{\sqrt{L_e L_d}} \cong \frac{B_{ue} A_d}{\sqrt{L_e L_d}} \tag{15}$$

where A_d is the effective area of the detection microcoil, and B_{ue} is the unitary field at center of the excitation microcoil

$$B_{ue} = \frac{2\sqrt{2}\mu_0}{\pi D_e} \tag{16}$$

where μ_0 is the vacuum permeability, and D_e is the side of the square excitation microcoil. Introducing the numerical values $D_e \cong 170$ μm, $D_{d,1} \cong 40$ μm, $D_{d,2} \cong 70$ μm, $A_d \cong D_{d,1}^2 + D_{d,2}^2 \cong 6500$ μm^2, $L_e \cong 500$ pH, $L_d \cong 300$ pH, we obtain a coupling factor $k \cong 0.11$.

The microwave magnetic field in the center of the square excitation microcoil $B_{mw,e}$ is

$$B_{mw,e} = B_{ue} I_e = \frac{2\sqrt{2}\mu_0}{\pi D_e} I_e \tag{17}$$

where the current I_e in the microwave current in the excitation microcoil. The microwave current in the excitation microcoil produces a microwave magnetic field which induces an electromotive force in the detection microcoil. This induced electromotive force determines in microwave current I_d in the detection microcoil, which produces a microwave magnetic field in the center of the detection microcoil equal to

$$B_{mw,d} = B_{ud} I_d = \frac{2\sqrt{2}\mu_0}{\pi} \left(\frac{1}{D_{d,1}} + \frac{1}{D_{d,2}} \right) I_d \tag{18}$$

From the circuit simulations of the complete system in Fig.S2a, the currents in the excitation and detection microcoils, with a 10 W source with V_s = 63 V, are $I_e \cong 110$ mA and $I_d \cong 75$ mA at $\omega_L \cong 2\pi \times 9.1$ GHz. Hence, from Eqs. 17 and 18, the magnetic field created by current in the excitation coil is $B_{mw,e} \cong 7.3$ G and the one created by the current I_d in the detection coil is $B_{mw,d} \cong 33$ G. Hence, the detection microcoil creates a microwave field which is about 5 times larger than the microwave field created by the excitation microcoil.

The simulations show also that the phase difference between the two magnetic field is smaller than $\pi/6$. Hence the effective microwave magnetic field (i.e., half of the total linearly polarized microwave field acting on the spin ensemble) is approximately given by

$$B_1 \cong \frac{1}{2}(B_{mw,e} + B_{mw,d}) \tag{19}$$

From this equation, considering the value obtained above for $B_{mw,e}$ and $B_{mw,d}$, the effective microwave field acting on the spin ensemble is $B_1 \cong 20$ G. This value is about a factor two larger than the one obtained experimentally measuring the Rabi nutation frequency, i.e., $B_1 \cong 9$ G (see main text), indicating that the model in Fig.S2a is relatively accurate.

C. Simulation of the receiver gain and noise

For the simulation of the receiver, we used the Virtuoso module of the Cadence simulator. The detection microcoil is simulated with the EM module of Advanced Design Systems (ADS) simulator. The obtained S-parameters are introduced in the Cadence simulator. All simulations are performed with VDD_{RX} =1.8 and 2.5 V.

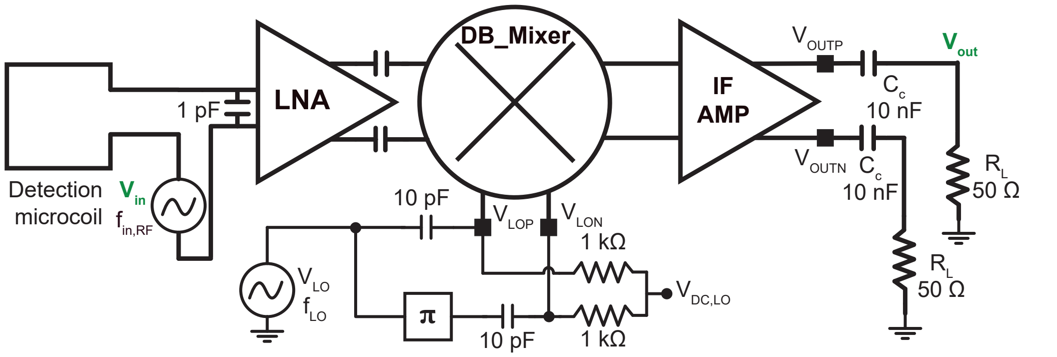

Figure S3: Receiver simulation setup. The detection microcoil is connected to the LNA with a 1 pF capacitor in parallel. The detection microcoil is modeled with the S-parameter file obtained by ADS simulations. V_{in} represents the electromotive force induced in the detection microcoil by the spin precession (i.e., the ESR signal). The local oscillator inputs V_{LOP} and V_{LON} are DC biased with two 1 kΩ resistors and a DC source $V_{DC,LO} \cong 1.5$ V. The LO signal amplitude is $V_{LO} \cong 3$ dBm, which is the optimum value for gain and noise.

Fig. S4a shows the overall gain of the receiver as a function of the microwave frequency $f_{in,mw}$, defined as $G = V_{out}/V_{in}$, at a constant offset frequency $f_{in,mw} - f_{LO} = 200$ MHz. Fig. S4b shows the overall gain of the receiver as a function of the offset frequency $f_{in,mw} - f_{LO}$, at a constant microwave frequency of $f_{in,mw}$ =9.1 GHz. Fig. S4c shows the input voltage noise spectral density (i.e., the equivalent noise spectral density at the detection coil ends) as a function of the microwave frequency $f_{in,mw}$, defined as $V_{n,in} = V_{n,out}/G$, at a constant offset frequency $f_{in,mw} - f_{LO} = 200$ MHz. Fig. S4d shows the input voltage noise spectral density (i.e., the equivalent noise spectral density at the detection coil ends) as a function of the offset frequency $f_{in,mw} - f_{LO}$,

at a constant microwave frequency $f_{in,mw} = 9.1$ GHz. Fig. S4e shows the output voltage noise spectral density as a function of the offset frequency $f_{in,mw} - f_{LO}$, at a constant microwave frequency $f_{in,mw} = 9.1$ GHz.

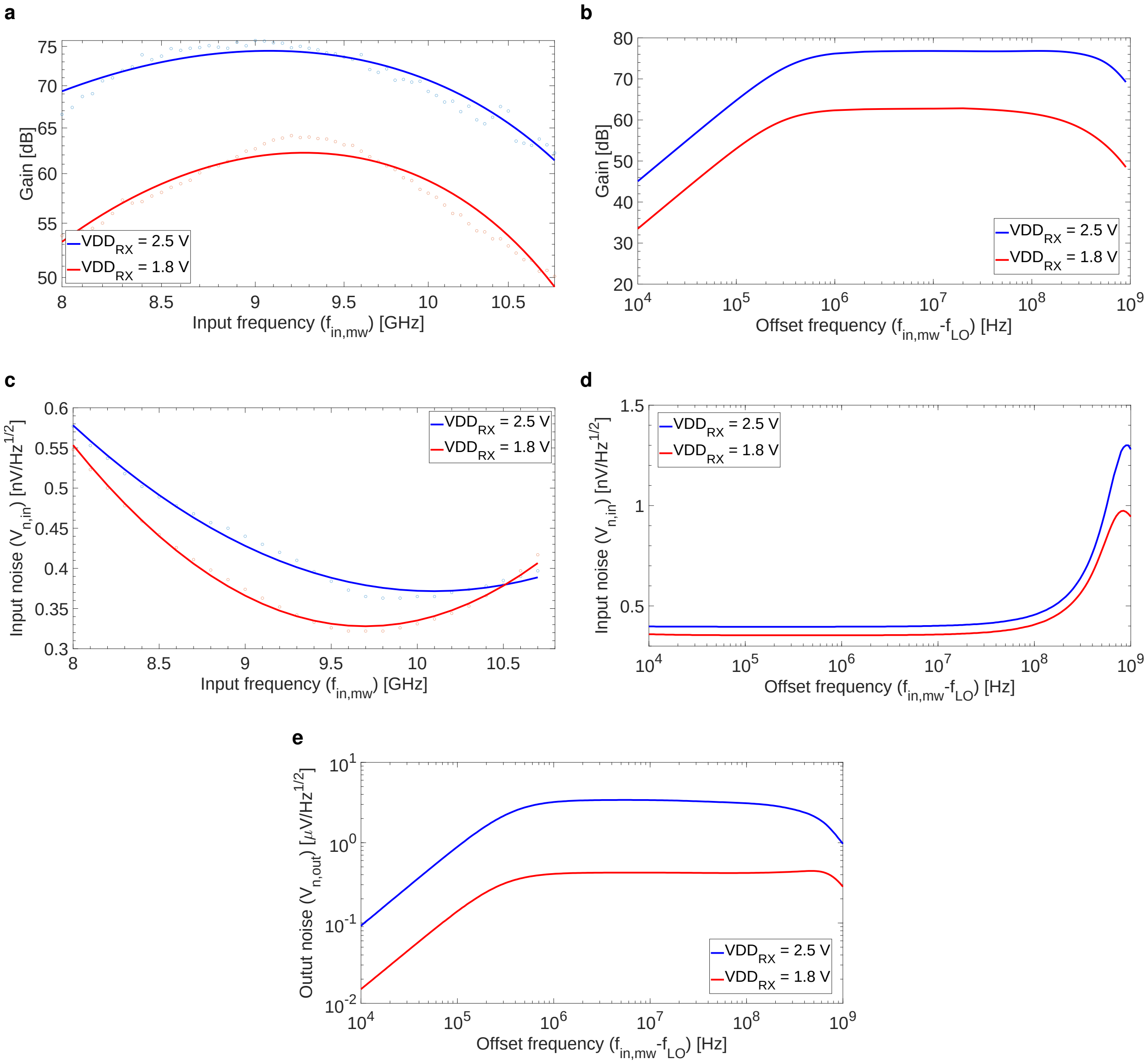

Figure S4: Receiver simulation results. **a** Overall gain of the receiver as a function of the microwave frequency $f_{in,mw}$, defined as $G = V_{out}/V_{in}$, at a constant offset frequency $f_{in,mw} - f_{LO} = 200$ MHz. **b** Overall gain of the receiver as a function of the offset frequency $f_{in,mw} - f_{LO}$, at a constant microwave frequency of $f_{in,mw}$ =9.1 GHz. **c** Input voltage noise spectral density (i.e., the equivalent noise spectral density at the detection coil ends) as a function of the microwave frequency $f_{in,mw}$, defined as $V_{n,in} = V_{n,out}/G$, at a constant offset frequency $f_{in,mw} - f_{LO} = 200$ MHz. **d** Input voltage noise spectral density (i.e., the equivalent noise spectral density at the detection coil ends) as a function of the offset frequency $f_{in,mw} - f_{LO}$, at a constant microwave frequency $f_{in,mw} = 9.1$ GHz. **e** Output voltage noise spectral density as a function of the offset frequency $f_{in,mw} - f_{LO}$, at a constant microwave frequency $f_{in,mw} = 9.1$ GHz.

References

[1] Ernst, R. R.; Bodenhausen, G.; Wokaun, A. *Principles of nuclear magnetic resonance in one and two dimensions*; Oxford university press, 1990.

[2] Dayan, N.; Ishay, Y.; Artzi, Y.; Cristea, D.; Driesschaert, B.; Blank, A. Electron spin resonance microfluidics with subnanoliter liquid samples. *Journal of Magnetic Resonance Open* **2020**, *2*, 100005.
